# Tracking tree demography and forest dynamics at scale using remote sensing

**DOI:** 10.1111/nph.20199

**Published:** 2024-10-18

**Authors:** Robin Battison, Suzanne M. Prober, Katherine Zdunic, Toby D. Jackson, Fabian Jörg Fischer, Tommaso Jucker

**Affiliations:** ^1^ School of Biological Sciences University of Bristol Bristol BS8 1TQ UK; ^2^ CSIRO Environment Canberra ACT 2601 Australia; ^3^ Biodiversity and Conservation Science Department of Biodiversity, Conservation and Attractions Kensington WA 6151 Australia

**Keywords:** competition, growth, LiDAR, mortality, recruitment, topography, tree crown delineation, wildfires

## Abstract

- Capturing how tree growth and survival vary through space and time is critical to understanding the structure and dynamics of tree‐dominated ecosystems. However, characterising demographic processes at scale is inherently challenging, as trees are slow‐growing, long‐lived and cover vast expanses of land.
- We used repeat airborne laser scanning data acquired across 25 km^2^ of semi‐arid, old‐growth temperate woodland in Western Australia to track the height growth, crown expansion and mortality of 42 213 individual trees over 9 yr.
- We found that demographic rates are constrained by a combination of tree size, competition and topography. After initially investing in height growth, trees progressively shifted to crown expansion as they grew larger, while mortality risk decreased considerably with size. Across the landscape, both tree growth and survival increased with topographic wetness, resulting in vegetation patterns that are strongly spatially structured. Moreover, biomass gains from woody growth generally outpaced losses from mortality, suggesting these old‐growth woodlands remain a net carbon sink in the absence of wildfires.
- Our study sheds new light on the processes that shape the dynamics and spatial structure of semi‐arid woody ecosystems and provides a roadmap for using emerging remote sensing technologies to track tree demography at scale.

## Introduction

Forest ecosystems face growing pressure on multiple fronts, from increasingly frequent and severe droughts and heatwaves, larger and more intense wildfires and storms, novel pests and pathogens, and human‐driven degradation (Senf *et al*., 2018; Canadell *et al*., 2021; Hammond *et al*., 2022; Turner & Seidl, 2023). Understanding how trees are responding to these novel disturbance regimes is critical if we are to forecast how forest dynamics will change over the coming century, and what implications this will have for biodiversity and carbon storage in these ecosystems (McDowell *et al*., 2020; Turner & Seidl, 2023). To achieve this, we need demographic information that allow us to infer and model changes in population dynamics at scale – data that capture how rates of tree growth, mortality and recruitment vary across both space and time (Coomes *et al*., 2014; Fisher *et al*., 2018; Kunstler *et al*., 2021; Needham *et al*., 2022b; Zuidema & van der Sleen, 2022). Ecologists have traditionally relied on networks of permanent field plots to estimate these demographic rates (Lines *et al*., 2010; Ruiz‐Benito *et al*., 2013; Kunstler *et al*., 2021; Needham *et al*., 2022b; Piponiot *et al*., 2022). However, while plot networks remain the gold standard to characterise community‐level dynamics, they have some inherent limitations when it comes to capturing variation in demographic rates across landscapes. Field surveys are incredibly labour‐intensive, meaning that most forest plots are small (0.1–1 ha) and cumulatively only cover a tiny fraction of the total forest area (< 0.01% even in best‐case scenarios; Yu *et al*., 2022; Holcomb *et al*., 2023). This makes it challenging to understand how demographic rates vary across environmentally heterogeneous landscapes and in response to large, infrequent disturbances.

Remote sensing offers an intuitive solution to this challenge of tracking large numbers of trees across broad spatial scales (Stovall *et al*., 2019; Brandt *et al*., 2020; Ma *et al*., 2023). In particular, technologies such as airborne laser scanning (ALS, or LiDAR) can be used to build highly accurate and detailed 3D models of both the forest canopy and the underlying terrain (≤ 1‐m resolution) that span thousands of hectares (Jucker, 2022; Lines *et al*., 2022). Unsurprisingly, ALS has become an integral tool for large‐area mapping of forest structure and biomass, and there is now a growing interest in using repeat ALS acquisitions to quantify forest dynamics at scale (Asner & Mascaro, 2014; Dalponte *et al*., 2019; Cushman *et al*., 2021; Dalagnol *et al*., 2021; Nunes *et al*., 2021; Jucker *et al*., 2023). To date, almost all this work has focussed on characterising dynamic processes occurring at a canopy level, such as those associated with the formation, expansion and closure of gaps (Wedeux *et al*., 2020; Cushman *et al*., 2021; Dalagnol *et al*., 2021; Nunes *et al*., 2021; Choi *et al*., 2023). However, in parallel, there has also been a push towards developing computational tools to detect individual tree crowns using ALS (Dalponte & Coomes, 2016; Ferraz *et al*., 2016; Cao *et al*., 2023). Benchmarking efforts have shown that algorithms can accurately segment and measure the crown dimensions of canopy‐dominant trees (Wang *et al*., 2016; Cao *et al*., 2023), particularly when applied to high‐resolution ALS data acquired in open canopy forests. As repeat ALS data become increasingly available, individual‐based methods provide a unique opportunity to characterise how rates of tree growth and mortality vary across size‐structured populations (Piponiot *et al*., 2022; Brandt *et al*., 2024) and whole landscapes (Duncanson & Dubayah, 2018; Stovall *et al*., 2019; Beese *et al*., 2022; Ma *et al*., 2023).

The appeal of individual‐based methods applied to large‐scale remote sensing data sets is not just in their ability to inventory huge numbers of trees. By capturing the 3D architecture of individual tree crowns, ALS data also provide a way to characterise tree growth along multiple axes, including height growth and lateral crown expansion (Lines *et al*., 2022). Vertical and horizontal crown growth are rarely recorded in field data, as they are much more complex and time‐consuming to measure than stem diameters. However, they are arguably much more ecologically meaningful when it comes to capturing whole‐plant growth strategies and how these vary with tree size, such as hypothesised shifts in biomass allocation away from height growth and towards crown expansion as trees approach maturity (Antin *et al*., 2016; Marziliano *et al*., 2019; Jucker *et al*., 2022; Laurans *et al*., 2024). Similarly, they are much more informative when it comes to understanding competitive interactions for light and space among neighbouring trees (Jucker *et al*., 2015; Taubert *et al*., 2015), as well as providing a way to quantify crown damage and dieback, which are strong predictors of tree mortality (Needham *et al*., 2022a; Zuleta *et al*., 2022). Another appeal of ALS data is that they contextualise the biotic and abiotic environment within which trees grow, such as their local competitive neighbourhood and topographic position within the landscape (Colgan *et al*., 2012; Swetnam *et al*., 2017; Beese *et al*., 2022; Ma *et al*., 2023). This provides an opportunity to not only quantify how demographic rates vary across the landscape but also ascribe this variation to underlying ecological drivers. Finally, a key selling point of individual‐based methods is that they are directly comparable to how we monitor forests on the ground and how we represent them in forest dynamics models. This provides an opportunity to bridge the gap between field and remote sensing forest monitoring programmes and can help reduce major sources of uncertainty in forest dynamics models, such as those associated with tree mortality (Hubau *et al*., 2020; McDowell *et al*., 2020; Pugh *et al*., 2020).

Here, we use data from two ALS surveys acquired 9 yr apart in Australia's Great Western Woodlands (GWW) to capture the height growth, crown expansion, crown dieback and mortality of individual trees across 2500 ha of old‐growth woodland habitat. These semi‐arid woodlands are an ideal testbed for using repeat ALS data to quantify tree demographic rates at scale, as they are dominated by a small number of eucalypt species that form sparsely populated stands of single‐stemmed trees. By developing a new pipeline for segmenting and matching tree crowns across ALS surveys, we were able to confidently identify and track the dynamics of 42 213 canopy‐dominant trees across this landscape. Using these data, we set out to:

1. Determine how growth and mortality rates vary with tree size across the whole population. This allowed us to quantify which cohorts contribute most to biomass gains and losses, as well as characterise how trees adjust their crown growth strategies as they increase in size.
2. Model how tree growth and mortality rates vary across the landscape in relation to fine‐scale topography and local neighbourhood competitive environment, so that we may better understand how demographic processes give rise to vegetation spatial patterns in dry forests.
3. Scale up tree‐level demographic rates to community‐level dynamics in aboveground biomass and canopy 3D structure. In doing so, we explored whether temporal changes in canopy 3D structure are predominantly driven by tree growth or mortality, and aimed to determine whether these old‐growth woodlands currently operate as a net carbon sink or source in the absence of large disturbances from wildfires.

## Materials and Methods

### Study system

The study was conducted at the GWW Terrestrial Ecosystem Research Network (TERN) SuperSite (30°11′29″S, 120°39′15″E; see Supporting Information Fig. S1), one of 16 intensively monitored TERN SuperSites distributed across Australia (Prober *et al*., 2023). Formerly a pastoral lease, since 2007, the land has been managed as a conservation area by the Western Australia Department of Biodiversity, Conservation and Attractions. The site is located on relatively flat terrain (430–475 m above sea level) with a mean annual temperature is 19°C and the mean annual rainfall is 260 mm. Soils are primarily deep red calcareous loams and clays over a deeply weathered regolith, with a hypersaline, acidic water table at > 20 m.

The landscape consists of a mosaic of different vegetation types, with the vast majority covered by old‐growth temperate eucalypt woodlands and the rest consisting of mulga scrub (*Acacia* sp.) and heathland (which collectively cover < 0.1% of the study area and were masked out of all analyses presented here; see Methods S1 for details). The woodland habitats, which are the focus of this study, are dominated by a small number of obligate‐seeder eucalypt species. The most abundant of these is *Eucalyptus salmonophloia* F.Muell., but *Eucalyptus salubris* F.Muell., *Eucalyptus transcontinentalis* Maiden and *Eucalyptus clelandiorum* Maiden are also found in relation to subtle variation in soils and topography. Wildfires are the major agent of disturbance in the region (Jucker *et al*., 2023) and are almost always stand replacing, as obligate‐seeder eucalypts are highly susceptible to fire and have almost no ability to resprout (Gosper *et al*., 2018). The site has not been affected by wildfires for several hundred years, and the woodlands are currently in an old‐growth successional stage. This is characterised by open canopy stands dominated by sparse, large, single‐stemmed trees, where localised stand dynamics are initiated by infrequent windthrows and floods, which are followed by subsequent recruitment in gaps (Gosper *et al*., 2018). Based on field data collected across three 1‐ha forest plots dominated by *E. salmonophloia*, *E. salubris* and *E. transcontinentalis*, typical ranges of tree density, basal area and aboveground biomass for old‐growth woodlands at this site are 15–34 trees ha^−1^, 4.9–5.1 m^2^ ha^−1^ and 37.9–45.3 Mg ha^−1^, respectively (field data available at: https://field.jrsrp.com).

### Airborne data acquisition and processing

**Fig. 1:**
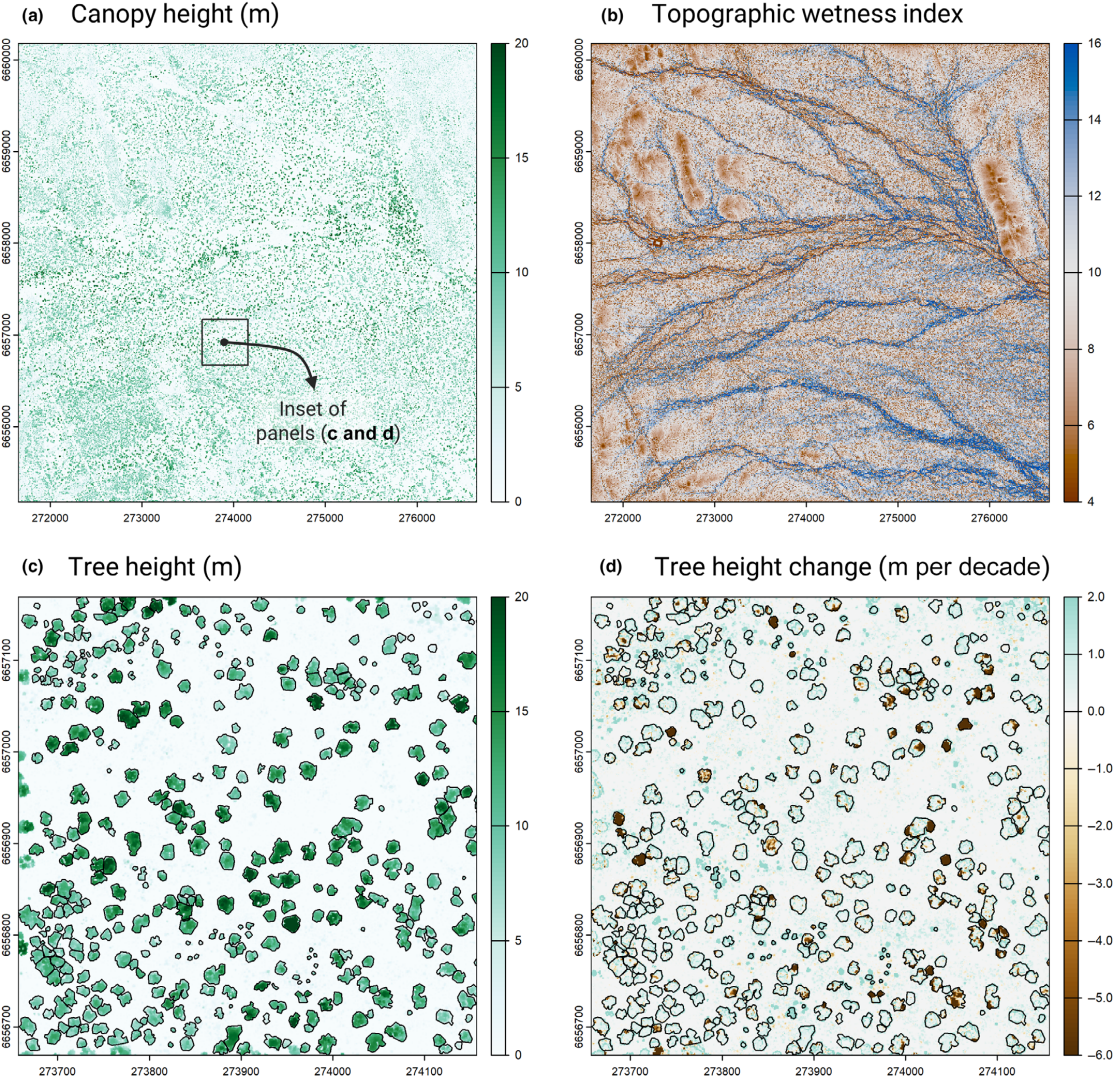
Tracking variation in tree demography across the Great Western Woodland TERN SuperSite using airborne laser scanning (ALS). (a) Canopy height model (CHM) of the entire study area (5 × 5 km) generated using the 2012 ALS data, while (b) shows variation in topographic wetness index (TWI) across the same extent, from which the major drainage channels of the site can be clearly seen in blue. (c) Corresponds to a 500 × 500 m section of the whole CHM (see black inset box in a), within which the crowns of individual canopy trees can be clearly seen. Changes in tree height between the two ALS surveys (2012 and 2021) are depicted in (d), where trees that died or underwent crown dieback are clearly visible in dark brown. The CHM has a resolution of 0.5 m, while TWI was calculated at 5‐m resolution. The coordinate reference system of the maps is GDA94 with a transverse Mercator projection (MGA zone = 51; EPSG = 28351; units = meters).

Airborne laser scanning data were acquired at two points in time over a 5 × 5 km (25 km^2^) square area centred on the GWW SuperSite (Fig. 1), first in May 2012 and then again exactly 9 yr later in May 2021. The 2012 data were collected by Airborne Research Australia (Parafield, Australia) using a motorised glider (HK36 TTC‐ECO; Diamond Aircraft Industries, Friesach, Austria) mounted with a RIEGL LMS‐Q560 scanner. Flights were conducted at 300 m aboveground with a scan angle of ±24°, resulting in a footprint size of 15.0 cm and an average pulse density of 21.4 pulses m^−2^. The 2021 data were acquired by Aerometrex (Glynde, Australia) using a Cessna 404 Titan mounted with a RIEGL VQ‐780ii scanner. Flights were conducted at 1100 m aboveground with a scan angle of ±30°, resulting in a footprint size of 19.8 cm and an average pulse density of 23.6 pulses m^−2^. During the second ALS survey, high‐resolution RGB imagery (20‐cm ground sample distance) were also acquired over the study area and provided as an orthomosaic. Georeferenced point clouds for both ALS surveys were provided in LAS 1.2 format, and all subsequent processing was performed using a combination of CloudCompare (https://www.danielgm.net/cc/), Qgis (https://qgis.org) and R (R Core Team, 2024) using the lidr (Roussel *et al*., 2020), terra (Hijmans *et al*., 2023), sf (Pebesma, 2018) and dynatopmodel packages (Metcalfe *et al*., 2015).

First, a statistical outlier removal filter implemented in CloudCompare was used to identify and remove outlier and duplicate points, following which we standardised the two acquisitions by removing any points with scan angles exceeding ±24°. Each point cloud was then classified into ground and nonground returns using the progressive morphological filter implemented in lidr, allowing a normalised point cloud to be produced for both timesteps. From these, a 0.5‐m resolution canopy height model (CHM) was then created for both 2012 and 2021 using the pit‐free algorithm implemented in the lidr package (Khosravipour *et al*., 2014). Alignment between the two CHMs was visually assessed and adjusted in Qgis, at which point we clipped the 2021 CHM to the extent of the 2012 CHM to only retain areas of overlap between the two flights (2500 ha in total). Finally, the 2021 classified point cloud was used to create a 5‐m resolution digital terrain model (DTM) of the study area using the triangular irregular network algorithm with Delaunay triangulation implemented in lidr.

### Automated tree crown detection and segmentation

Several algorithms have been developed in recent years to automatically detect and delineate individual tree crowns in ALS‐derived CHMs (Eysn *et al*., 2015; Dalponte & Coomes, 2016; Aubry‐Kientz *et al*., 2019; Cao *et al*., 2023). To determine which of these was most suitable for our study system, we began by manually delineating the crowns of all trees taller than 4 m and with a crown area > 9 m^2^ within a 1000 × 500 m (50 ha) section of the 2012 CHM (*n* = 797 manually delineated crowns in total). Using this as a benchmark, we first compared four alternative crown delineation algorithms implemented in the lidr package (*dalponte2016*, *li2012*, *silva2016* and *watershed*) based on several complementary criteria, including: (1) total number of crowns detected, (2) mean crown area of correctly matched crowns, (3) percentage of correctly segmented, oversegmented and omitted crowns, (4) Intersection over Union (IoU, %) of manually delineated and algorithm‐segmented crown polygons and (5) and an F_1_ score obtained by first classifying crowns as either correctly segmented or not using IoU > 50% as a cut‐off and then calculating precision and recall as described in Cao *et al*. (2023). F_1_ scores vary between 0 and 1, with a value of 0.7 generally being considered good.

Consistent with previous studies (Eysn *et al*., 2015; Aubry‐Kientz *et al*., 2019), we found that *dalponte2016* performed very well both in absolute terms and relative to the other algorithms we tested (78% correctly segmented crowns; mean IoU = 75%; F_1_ score = 0.92; Table S1; Fig. S2). However, despite its robust overall performance, the default implementation of *dalponte2016* exhibited a relatively high degree of oversegmentation (13%), especially for large trees (35% for trees with a crown area > 200 m^2^; Fig. S2). To avoid this having an undue effect on our results, we developed a new implementation of the *dalponte2016* routine specifically designed to address this issue of oversegmentation of large trees. This approach is described in detail in Methods S2, but briefly it involves running the crown segmentation in two stages: first using a broad search window to accurately segment the crowns of large trees (Cao *et al*., 2023), followed by a second pass with a smaller search window to detect any crowns missed in the initial step. Both search windows were defined based on allometric constraints between crown width and height, which we modelled using data from the 797 manually segmented trees (Fig. S3). This two‐stage approach considerably improved the performance of the algorithm, increasing overall accuracy to 82% and reducing oversegmentation to 5% (Table S1; Fig. S4). This new implementation of the *dalponte2016* algorithm was applied to the entire 2012 and 2021 CHMs to segment all trees taller than 4 m and with a crown area > 9 m^2^ within the study area.

### Estimating rates of tree height growth, crown expansion, mortality and biomass change

#### Changes in tree height and crown area

To robustly estimate changes in the height and crown area of individual trees, we developed a routine for matching tree crowns detected across both ALS surveys. We began by checking whether the crown polygon of a tree delineated in 2012 also contained the centroid of a crown detected in 2021, keeping only those that did. A crown that was only delineated in 2012 would indicate either a tree that died or underwent substantial crown dieback between surveys (or possibly one that was mistakenly omitted by the segmentation routine, but these are a minority; Table S1). Then, to retain only high‐quality matches, we removed any instances in which a crown delineated in one of the two ALS surveys contained two or more crown centroids in the other time period. This step was designed to detect instances in which the delineation algorithm mistakenly oversegmented a tree into multiple crowns. While our analysis of the training data suggests oversegmentation errors were infrequent (5%; Table S1), retaining them would bias our estimates of tree growth by introducing extreme values of height and crown area change.

This left us with a subset of crowns for which we have a high degree of confidence that the same tree was detected at both time points. From these, we further removed any trees that underwent a ≥ 30% decrease in height and/or crown area, which were classified as dead or having undergone severe dieback (Duncanson & Dubayah, 2018; Ma *et al*., 2023). All remaining matched crowns were used to estimate changes in individual tree height (m per decade) and crown area (m^2^ per decade) between the two ALS acquisitions. Tree height was defined as the maximum value of the CHM within the crown polygon, which is analogous to how it is typically measured in field surveys (Jucker *et al*., 2022); although we note that taking the mean value of the CHM yielded very similar results (Fig. S5).

#### Tree mortality and crown dieback

Two complementary approaches were used to identify trees that died or underwent pronounced crown dieback (hereafter treated as a single, combined category referred to as ‘mortality’) between the two ALS surveys (Fig. 1d). First, we used the set of matched crowns described above to screen for trees that decreased in height and/or crown area by ≥ 30% throughout the study period and classified these as dead or having experienced severe dieback (Duncanson & Dubayah, 2018; Ma *et al*., 2023). Second, to capture trees that completely disappeared between the two surveys (i.e. those identified only in the 2012 scan), we overlayed all unmatched crowns from 2012 onto both CHMs and measured their change in height from one time point to the next. Applying the same threshold as before, all trees that decreased in height by ≥ 30% were classified as dead.

Note that the inverse approach could be used to estimate rates of tree recruitment into the population (i.e. trees exceeding a height ≥ 4 m and crown area ≥ 9 m^2^ only in 2021). However, we chose not to implement this into our analysis, as trees in these smaller size classes exhibited the highest rates of both height growth and mortality (see the Results section). As a result, over the course of the 9‐yr period separating the two ALS scans, trees could have surpassed the minimum size threshold only to then die before the second survey took place, causing true recruitment rates to be substantially underestimated.

#### Aboveground biomass stocks and changes

To estimate the aboveground biomass (AGB, in kg) of individual trees detected in the 2012 and 2021 ALS surveys, we followed a two‐stage approach based on the framework developed in Jucker *et al*. (2017). First, we estimated each tree's diameter at breast height (DBH, in cm) based on its height (*H*, in m) and crown diameter (CD, in m) using the following allometric model: $\mathrm{DBH}=0.519\times {\left(H\times \mathrm{CD}\right)}^{0.890}\times {\mathrm{CF}}_{\mathrm{DBH}}$, where CF_DBH_ = 1.002 and corresponds to the Baskerville correction factor. *H* and CD were derived from the segmented tree crown polygons, with CD calculated from crown area assuming a circular crown. This allometric model was developed using data from angiosperm trees growing in woodland and savanna ecosystems of Australasia (Jucker *et al*., 2017).

We then estimated each tree's aboveground biomass using an allometric model developed specifically for eucalypt trees in Australia (Paul *et al*., 2016): $\mathrm{AGB}=0.133\times {\left(\mathrm{DBH}\right)}^{2.375}\times {\mathrm{CF}}_{\mathrm{AGB}}$, where CF_AGB_ = 1.067. Each tree's aboveground biomass change was then calculated as the difference in AGB between the two ALS surveys (expressed in kg per decade). For these calculations, trees that were classified as having died or undergone severe crown dieback were assumed to have lost all their biomass (AGB_2021_ = 0).

### Characterising size‐dependent rates of tree growth, mortality and biomass change

To build an overall picture of population‐level trends in tree demographic rates across the landscape and how they vary with tree size, we aggregated the tree‐level data on height growth, crown expansion and mortality according to size class. As a measure of initial tree size, for each tree, we used the data from 2012 to calculate the product of its height and crown diameter (*H* × CD, in m^2^), which previous work has shown to be strongly related to a tree's total aboveground biomass (Jucker *et al*., 2017; Ma *et al*., 2023). Trees were then assigned to one of 10 size classes by binning values of *H* × CD into logarithmic bins of equal‐width (logarithmic binning was chosen to better capture the right‐skewed distribution of the data). Each size class was represented by at least 1700 trees (range = 1744–7255 trees per size class). To test how rates of tree height growth and crown expansion vary with tree size, we fit ANOVAs to compare mean values of both growth metrics across the 10 size classes. Similarly, to characterise how tree mortality rates change with tree size, we used logistic regression (generalised linear model with binomial errors and logit link) to estimate the probability of a tree being alive (0) or having died/experienced severe dieback (1) by the time of the second ALS survey for each of the 10 size classes.

To understand how variation in growth rates and mortality translate to changes in aboveground biomass, for each size class, we calculated the total biomass gains of trees that survived, the biomass losses of those that died and the net biomass change between the two ALS surveys (each expressed in Mg ha^−1^ per decade). For this analysis, trees were binned into 10 percentile size classes (rather than equal‐width bins) so that each size class contained the same number of trees (*n* = 4221). This allowed us to directly compare the contribution of each size class to the aboveground biomass dynamics of the whole population.

### Drivers of landscape‐scale variation in tree demographic rates

To determine what processes shape variation in tree demographic rates across the landscape, we used generalised additive models (GAMs) to model variation in height growth, crown expansion, and mortality and dieback as a function of several intrinsic and extrinsic factors. First, to capture how growth allocation to height, crown expansion and risk of mortality vary in relation to tree size and life stage (Ruiz‐Benito *et al*., 2013; Jucker *et al*., 2022), we included initial tree size (i.e. *H* × CD in 2012) as a model predictor. Next, to account for the effects of neighbourhood competition on tree growth and mortality (Canham *et al*., 2004; Ma *et al*., 2023), we used the 2012 CHM to calculate a metric of local competitive environment for each tree. Specifically, a 25‐m buffer was added around the perimeter of each delineated crown, within which we calculated the mean height of the CHM as a proxy of local stand basal area (Asner & Mascaro, 2014). In semi‐arid ecosystems such as the GWW, competition among neighbouring trees is as much (if not more) for water as it is for light, which is why we chose a size‐symmetric measure of competition that directly reflects the basal area of neighbouring trees (Forrester *et al*., 2022; Ma *et al*., 2023). Third, to test how demographic rates vary in relation to local topography (Jucker *et al*., 2018), we used the dynatopmodel package to calculate topographic wetness index (TWI) at 5‐m resolution from the DTM (Fig. 1b) and then assigned a value of TWI to each tree based on their location. TWI describes how water flows and accumulates across the landscape, thus providing an indicator of local soil water and nutrient availability for plants (Kopecký *et al*., 2021). Finally, to control for differences in pulse density between ALS surveys, which could otherwise bias estimates of growth and mortality (Roussel *et al*., 2017), we included the difference in pulse density between the two scans as a model predictor (Fig. S6). This accounts for the fact that we would overestimate growth rates of trees scanned with a higher pulse density in 2021 simply by virtue of the more intense sampling of their crowns (and vice versa for 2012; Fischer *et al*., 2024).

Generalised additive models were fit using the mgcv package in R (Wood, 2011) and were chosen specifically to capture the fact that both tree growth and mortality vary nonlinearly with tree size (Coomes *et al*., 2012). However, as we had no *a priori* reason to expect that the effects of TWI, competition or pulse density on tree growth and mortality would be nonlinear, all other model predictors were fit as linear parametric terms (Table S2). To determine the optimal degree of smoothing for the tree size term, GAMs were fit using restricted maximum likelihood estimation (REML). Individual tree height growth and crown expansion rates were modelled assuming a Gaussian distribution, while probability of mortality was modelled with binomial errors and a logit link. To allow direct comparisons between model coefficients, all linear predictors were scaled and centred to have a mean of 0 and a SD of 1 before model fitting. Model residuals were tested for spatial autocorrelation, but we found little or no evidence of this (Fig. S7).

### Mapping spatial variation in demographic rates and its impacts on biomass dynamics and canopy 3D structure

To characterise spatial variation in demographic rates across the landscape, we mapped mean rates of tree height growth, crown expansion and tree mortality (% of stems that died or underwent severe crown dieback) across the GWW SuperSite. Individual tree‐level data were aggregated and mapped at a 1‐ha resolution across the 25 km^2^ study area (*n* = 2491 ha, after masking out 9 ha dominated by mulga habitat). This allowed us to visualise spatial patterns in demographic rates across the landscape and quantify the degree to which growth and mortality rates covary spatially. Then, to better understand how tree growth and mortality combine to drive aboveground biomass dynamics, we used the same approach to also map net changes in aboveground biomass across the landscape. In doing so, we explored whether biomass dynamics are more strongly constrained by tree growth or mortality, and assessed whether these old‐growth woodlands are currently operating as a net carbon sink or source.

To complement these individual tree‐based estimates of biomass dynamics, we also calculated two independent measures of changes in canopy 3D structure, which we derived directly from the CHMs at 1‐ha resolution. The first was a measure of change in canopy cover at 4‐m aboveground between 2021 and 2012 (Δ_cover_, expressed as % per decade). Canopy cover was defined as the proportion of CHM pixels exceeding 4 m in height, with this threshold chosen to match that used to include trees in our segmentation routine. The second was an estimate of change in canopy volume (Δ_vol_, expressed as m^3^ per decade) over the same period, where canopy volume was calculated by multiplying the height of each CHM pixel by its area and then summing across the 1‐ha grid. Note that Δ_vol_ is equivalent to calculating changes in mean canopy height (with a 1 m gain in canopy height corresponding to +10 000 m^3^ in canopy volume). By quantifying the degree of spatial correlation between aboveground biomass change and Δ_cover_ and Δ_vol_, we were able to determine how well changes in canopy 3D structure can be inferred when the underlying demographic rates are known.

## Results

Across the GWW SuperSite, we identified a total of 53 639 tree crowns in 2012 and 55 332 in 2021 (21.5 and 22.1 trees ha^−1^, respectively). Of these, we were able to confidently match 42 213 tree crowns across the two surveys, 3801 of which were classified as having died or experienced severe dieback between the two ALS surveys (11.0% per decade). In 2012, matched trees had a mean height of 10.9 m (interquartile range = 8.0–13.1 m; 99^th^ percentile = 20.9 m) and a mean crown area of 125.3 m^2^ (interquartile range = 46.8–175.5 m^2^; 99^th^ percentile = 450.0 m^2^). By 2021, trees that survived had grown an average of 0.20 m per decade in height (interquartile range = 0.07–0.37 m per decade) and expanded their crowns by 13.7 m^2^ (interquartile range = 0.3–25.0 m^2^ per decade). Estimated height growth rates were noticeably larger than instrument measurement errors, which we estimated by comparing the 2012 and 2021 DTMs (root mean square error = 0.03 m).

### Size‐dependent variation in tree demographic rates and biomass dynamics

**Fig. 2:**
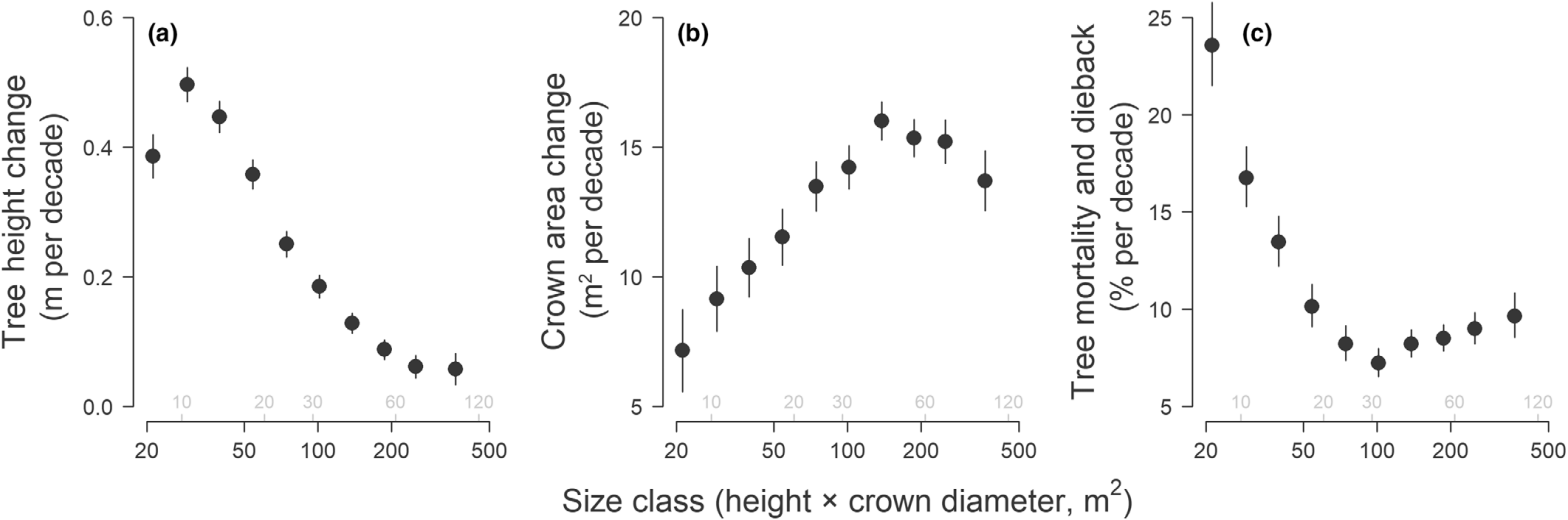
Variation in mean (a) tree height growth, (b) crown area growth and (c) tree mortality and crown dieback rates among tree size classes. Error bars correspond to 95% confidence intervals of the mean for each size class. Tree size was expressed as the product of tree height and crown diameter, which we grouped into 10 equal‐width bins. For comparison, we also provide corresponding estimates of stem diameter (in cm) for trees in different crown size classes, shown in grey at the bottom of each panel. Each size class in the analysis is represented by at least 1700 trees each. Trees were classified as dead or having undergone dieback if they exhibited a decrease in height and/or crown area of ≥ 30% between the two airborne laser scanning surveys.

**Fig. 3:**
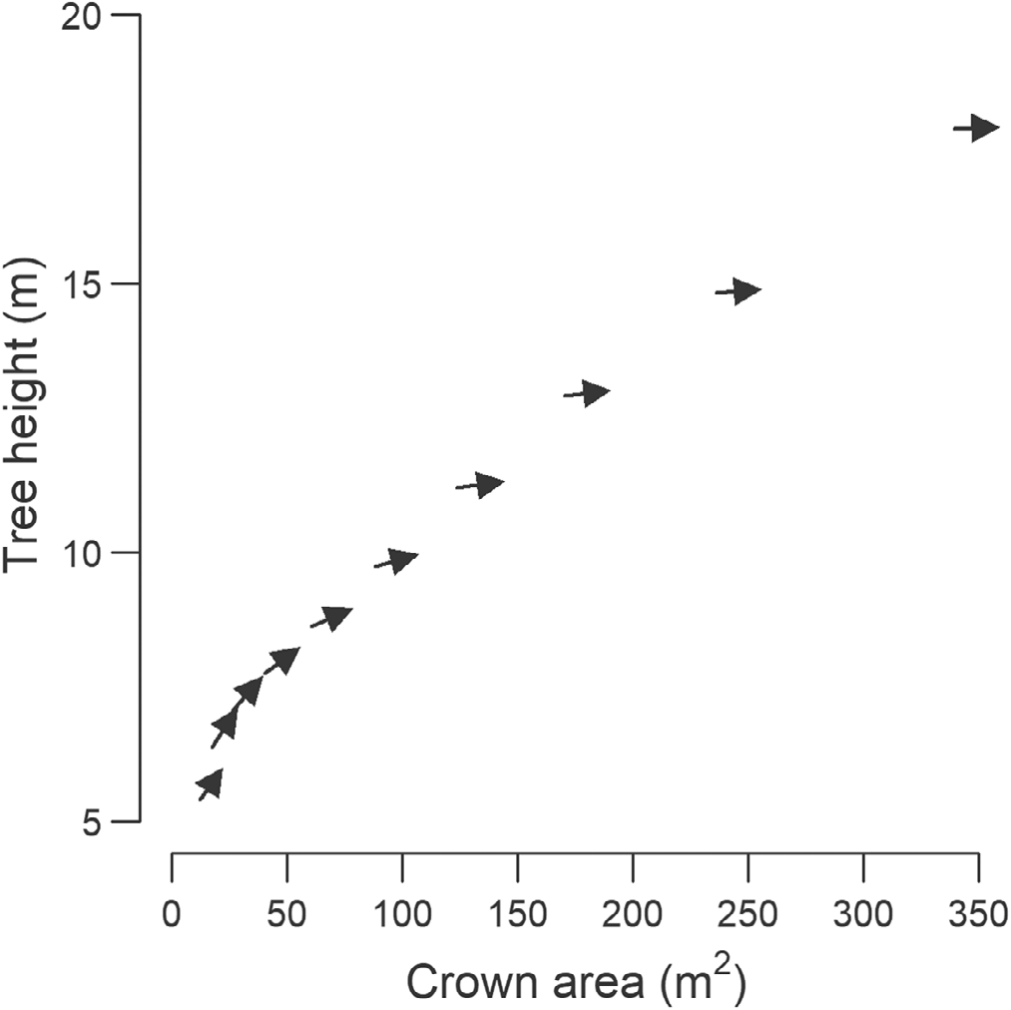
Changes in allocation to height and crown area growth with tree size. Each arrow illustrates the growth trajectory of trees within a size class, with the beginning of the arrow corresponding to the mean height and crown area of trees in 2012 and the end of the arrow indicating the size reached by the time of the second survey in 2021. Arrows pointing upwards at a 45° angle indicate trees growing both in height and crown area, while ones pointing horizontally to the right denote trees that expanded their crowns but remained unchanged in terms of height. Trees were grouped into 10 equal‐width size class bins, where size was defined as the product of tree height and crown diameter. Each size class in the analysis is represented by between 1744 and 7255 individual trees.

Mean rates of tree height growth, crown expansion and mortality all varied considerably with tree size (Fig. 2). Tree height growth peaked in small trees (0.50 m per decade) and decreased progressively thereafter to just 0.06 m per decade in the largest size classes (Fig. 2a). By contrast, crown area showed the opposite pattern (Fig. 2b), with mid‐sized and larger trees investing more than twice as much as small trees in crown expansion (13.7–16.0 and 7.2 m^2^ per decade, respectively). Together, these opposing growth trends highlight how trees progressively shift their woody biomass allocation away from tree height growth and towards crown expansion as they become larger (Fig. 3). As for risk of mortality and severe crown dieback, this initially decreased sharply with tree size before plateauing to *c*. 7.2–9.3% per decade in medium and large‐sized trees (Fig. 2c). Overall, trees in the smallest size class were around three times as likely to die or undergo dieback (23.6% per decade) as those in the mid and larger size classes.

**Fig. 4:**
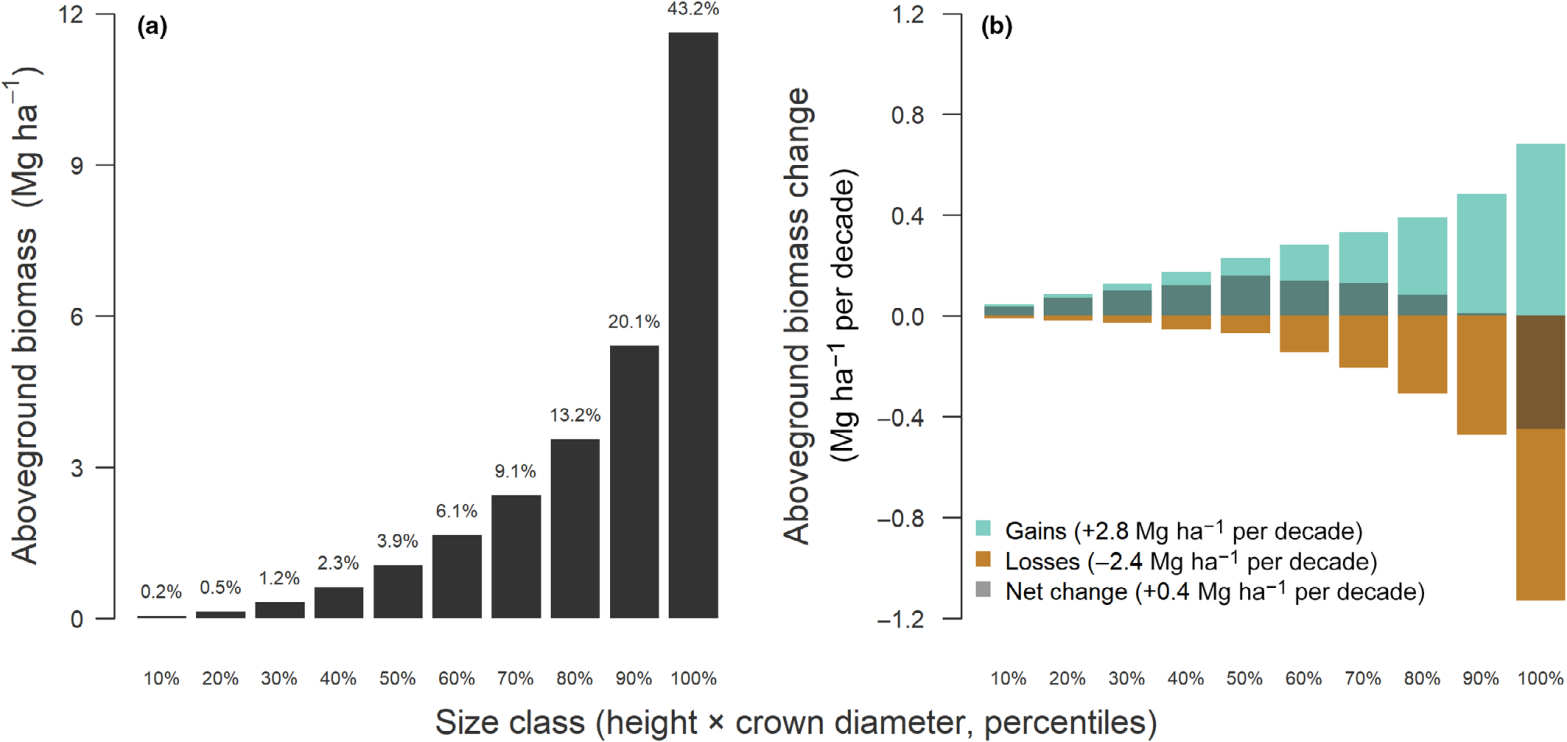
Variation of (a) aboveground biomass stocks and (b) aboveground biomass dynamics among tree size classes. Trees were binned into 10 percentile size classes based on the product of their height and crown diameter so that every bin contains the same number of trees. In (a), each bar corresponds to the cumulative aboveground biomass of trees belonging to a particular size class at the time of the first survey in 2012. The values reported at the top of each bar denote the proportion of the total aboveground biomass stored in each size class, averaged across the entire study site (mean total aboveground biomass = 26.9 Mg ha^−1^). In (b), bars indicate both biomass grains resulting from tree growth (green) and losses associated with mortality and crown dieback (brown) across each size class. The darker shaded area of each bar corresponds to the net change in aboveground biomass (i.e. gains – losses) for each size class, which was positive (dark green) for all expect the largest size class of trees (dark brown). Values reported in the legend of (b) are estimated total gains (+2.8 Mg ha^−1^ per decade), losses (−2.4 Mg ha^−1^ per decade) and net change in aboveground biomass (+0.4 Mg ha^−1^ per decade) across all size classes combined.

When growth and mortality rates were translated to changes in aboveground biomass, we again observed strong differences among size classes (Fig. 4). In terms of stocks, most of the aboveground biomass is stored in large trees, with the largest 10% of trees accounting for 43.2% of the aboveground biomass, while the smallest 50% only make up 8.2% (Fig. 4a). A similar picture emerged when looking at rates of biomass gains and losses over time, both of which increased continuously with tree size (Fig. 4b). Across the whole population, aboveground biomass gains associated with growth (+2.8 Mg ha^−1^ per decade) were greater than losses driven by mortality and crown dieback (−2.4 Mg ha^−1^ per decade). Consequently, net changes in aboveground biomass were positive (+0.4 Mg ha^−1^ per decade), suggesting that the woodland as a whole is currently operating as a net carbon sink, albeit a weak one. When broken down by size class we found that it was medium‐sized trees that contributed most to this net sink (net aboveground biomass change of trees in the 30–70% size percentile = +0.65 Mg ha^−1^ per decade). By contrast, a combination of decreasing growth rates and progressively greater losses in biomass linked to the mortality of large‐stature trees meant that contributions to net biomass gains declined in the larger size classes and even became negative in the largest size class (−0.45 Mg ha^−1^ per decade).

### Drivers of landscape‐scale variation in tree demographic rates

**Fig. 5:**
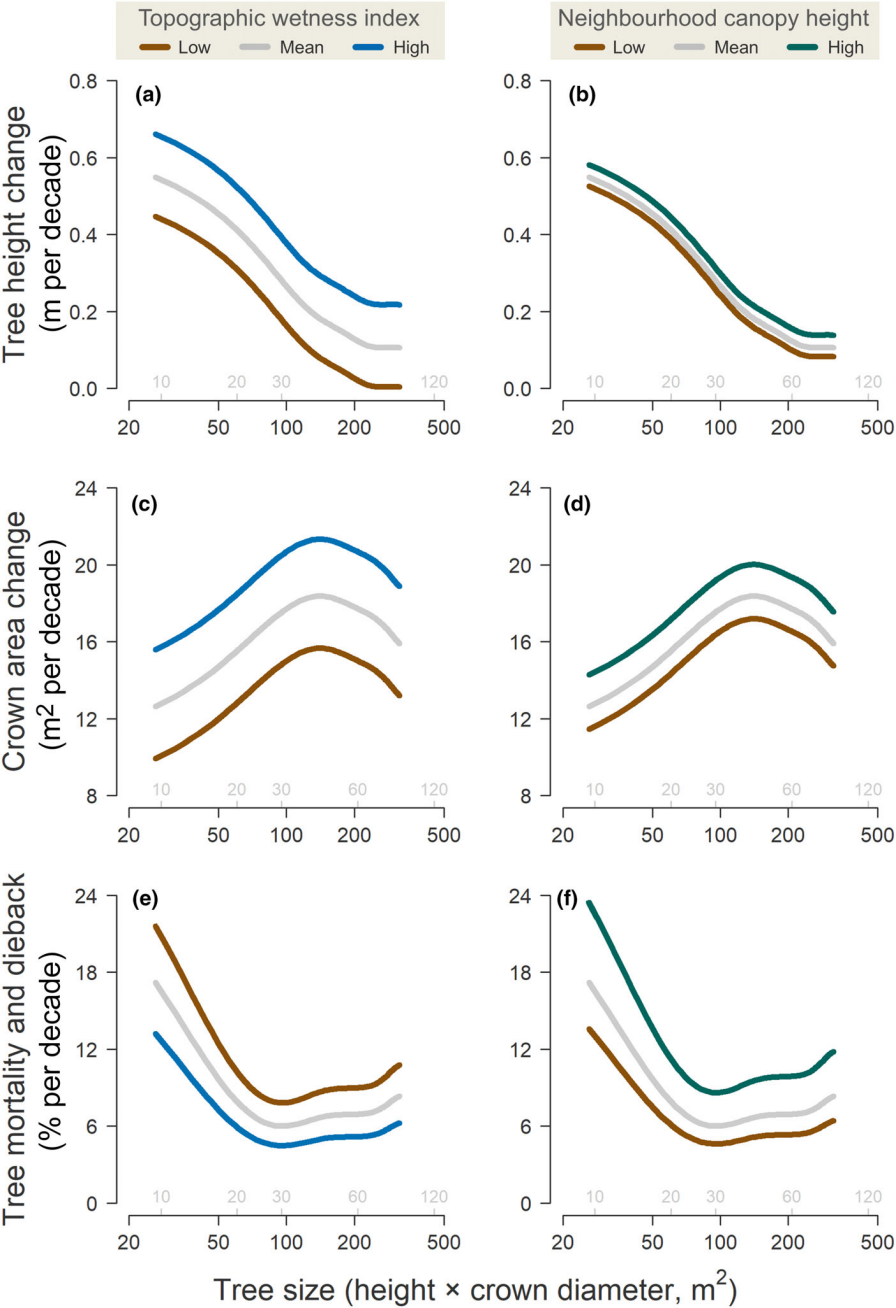
Influence of topographic wetness index (TWI, lefthand panels) and neighbourhood canopy height (righthand panels) on predicted rates of (a, b) tree height change (c, d) crown area change and (e, f) risk of mortality and crown dieback. Curves correspond to predicted values obtained from the generalised additive models described in Supporting Information Table S2, where pulse density difference was set to 0 and tree size was allowed to vary from the 5^th^ to 95^th^ percentile of the data. For TWI, we predicted values of each demographic rate for trees growing at low (5^th^ percentile, brown curves), mean (grey curves) and high (95^th^ percentile, blue curves) values of TWI, while keeping neighbourhood canopy height fixed at its mean value. The same procedure was used to predict variation in demographic rates in relation to neighbourhood canopy height. For comparison, we also provide corresponding estimates of stem diameter (in cm) for trees in different crown size classes, shown in grey at the bottom of each panel.

Topography and local competitive environment emerged as important drivers of variation in tree growth and mortality across the landscape, each exerting an effect that was comparable in magnitude to that of tree size (Fig. 5; Table S2). Both tree growth and probability of survival increased with TWI. After accounting for the effects of tree size, neighbourhood height and pulse density, predicted height growth was around four times higher for trees in areas of the landscape with high TWI (0.30 m per decade at 95^th^ percentile of TWI) than for ones with low TWI (0.08 m per decade at 5^th^ percentile of TWI). Similarly, rates of crown expansion increased from 15.7 to 21.3 m^2^ per decade and probability of mortality decreased from 8.7% to 5.0% per decade when transitioning from low to high TWI (brown vs blue curves in the left‐hand panels of Fig. 5).

Tree growth was also faster in taller stands, especially in the case of crown expansion, which for the average surviving tree increased from 17.2 to 20.0 m^2^ per decade when comparing trees growing in short (5^th^ percentile) vs tall (95^th^ percentile) neighbourhoods (brown vs green curves in Fig. 5d). However, the opposite was true for mortality, which nearly doubled for trees in tall neighbourhoods relative to ones in short, open stands (9.5% vs 5.1% per decade; Fig. 5f). Finally, our analysis also confirmed the importance of statistically controlling for local differences in pulse density among scans, as we found that height growth, crown expansion and survival were all overestimated in trees sampled at higher densities in 2021 (Table S2).

### Landscape‐scale variation in tree demographic rates and its impacts on biomass dynamics and canopy 3D structure

**Fig. 6:**
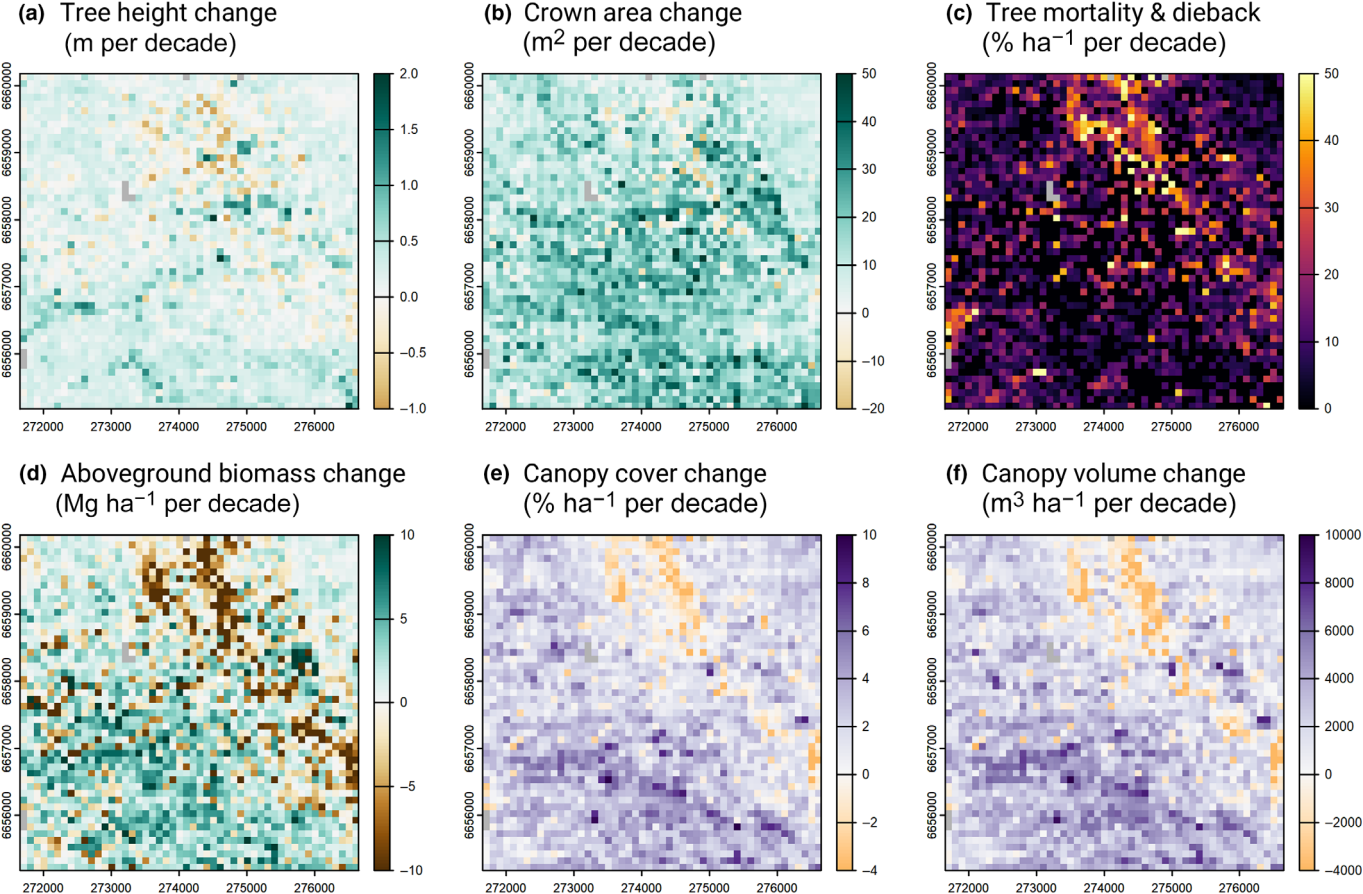
Spatial variation in rates of (a) tree height change, (b) crown area change, (c) tree mortality and dieback, and (d) aboveground biomass change across the Great Western Woodland SuperSite between 2012 and 2021. (a–d) Generated by aggregating the individual tree‐level data at 1‐ha resolution across the 5 × 5 km study area (*n* = 2491 ha, after masking out 9 ha dominated by mulga habitat, shown in grey in the maps). For (a–b), only trees that were classified as alive at both census periods were used to generate the maps. For comparison, rates of (e) canopy cover change (defined as % ground cover at 4 m aboveground) and (f) canopy volume change across the site are also shown. These two canopy‐level metrics were calculated directly from the 2012 and 2021 canopy height models, without delineating and tracking changes in individual tree crowns through time. The coordinate reference system of the maps is GDA94 with a transverse Mercator projection (MGA zone = 51; EPSG = 28351; units = meters).

We observed considerable spatial variation in tree demographic rates aggregated at 1‐ha scale (Fig. 6a–c), with tree height change ranging from −0.25 to 0.66 m ha^−1^ per decade, crown area changes from 0.4 to 33.2 m^2^ ha^−1^ per decade and probability of mortality from 0 to 33.3% ha^−1^ per decade (values are 5^th^ and 95^th^ percentile of the data shown in Fig. 6a–c). Rates of tree height growth and crown expansion were moderately positively correlated with one another (*ρ* = 0.31). By contrast, we found only a very weak negative relationship between mortality and both height and crown area growth (*ρ* = −0.11 and −0.15, respectively), indicating that these demographic axes were largely decoupled at the landscape scale.

On average, aboveground biomass stocks increased by 0.4 Mg ha^−1^ per decade (+1.4%), from 26.9 Mg ha^−1^ in 2012 to 27.3 Mg ha^−1^ in 2021. However, both biomass stocks (6.9–54.3 Mg ha^−1^) and rates of net biomass change over time (−9.2 to 6.5 Mg ha^−1^ per decade) varied considerably across the landscape (Fig. 6d). Areas that experienced faster rates of tree height growth (*ρ* = 0.32) and crown expansion (*ρ* = 0.44) also had the greatest increases in aboveground biomass, but in general spatial variation in biomass change was most strongly tied to rates of tree mortality (*ρ* = −0.61).

Over the same period, we also found substantial variation in rates of canopy cover (Δ_cover_) and volume change (Δ_vol_) across the landscape (Fig. 6e,f). In 2012, mean canopy cover at 4‐m aboveground was 24.0% ha^−1^ and mean canopy volume was 25 172 m^3^ ha^−1^. On average, by 2021, both metrics had increased by *c*. 7.8% across the landscape, with canopy cover reaching 25.9% ha^−1^ and canopy volume 27 130 m^3^ ha^−1^. But there were also substantial portions of the landscape where both canopy cover and volume decreased relative to 2012. Specifically, we observed a very distinct and large diagonal track (*c*. 3 km long and 200 m wide) in the northeast corner of the site where most of these losses were concentrated (see orange pixels in Fig. 6e,f). This same pattern is also clearly visible in the tree‐level growth, mortality and biomass change maps (Fig. 6a–d), indicating that these two independent approaches to quantifying canopy dynamics are spatially coherent. Specifically, we found that both Δ_cover_ and Δ_vol_ were closely correlated with estimated rates of aboveground biomass change across the landscape (*ρ* = 0.63 and 0.71, respectively).

## Discussion

### Gaining new perspective on tree demography using remote sensing

Using repeat ALS data, we were able to quantify rates of tree height growth, crown expansion, dieback and mortality across tens of thousands of trees and map their variation across an entire landscape. This new approach to tracking tree demography from above using remote sensing enabled us to gain unique insights into the process that shape forest dynamics at scale in a way that would have been impossible from the ground.

From a resource allocation perspective, our results revealed a clear pattern of trees shifting their growth strategies as they became larger (Figs 2, 3). When small, trees initially invested primarily in height growth, presumably to establish their crowns in the canopy (Laurans *et al*., 2024) and as a strategy to reduce ladder fuels and fire spread (Gosper *et al*., 2018). But as they grew larger, trees began progressively increasing their growth allocation towards crown expansion, with height growth decreasing to almost nothing in the largest size classes. This strategy appears to be common across forest types (Antin *et al*., 2016; Marziliano *et al*., 2019) but makes particularly good sense in open and dry environments such as the GWW. Here being too tall needlessly increases the risk of drought‐induced embolism (Olson *et al*., 2018) as there is little to be gained from a light competition perspective. Instead, a more efficient strategy for maximising light interception is to expand one's crown outwards (Jucker *et al*., 2024). Similar growth patterns have also been observed in African savannas, although here the initial investment in height growth has been attributed not just to escaping fire risk but also herbivory (Moncrieff *et al*., 2011). More generally, by characterizing how growth allocation strategies vary with tree size, we can better understand how static allometric constraints between tree height and crown size emerge as a result of dynamic growth processes (Fig. 3; Fischer *et al*., 2019; Jucker *et al*., 2022; Lines *et al*., 2022; Laurans *et al*., 2024). This is a critical step for building more realistic models of forest dynamics that accurately represents the 3D structure of forest canopies (Fischer *et al*., 2020; Jucker, 2022).

From a demography perspective, one the biggest contributions that remote sensing technologies such as repeat ALS can offer is the ability to comprehensively characterise rates and patterns of tree mortality (Duncanson & Dubayah, 2018; Stovall *et al*., 2019; Ma *et al*., 2023). Growing evidence suggests that rates of tree mortality are on the rise globally (Senf *et al*., 2018; McDowell *et al*., 2020; Hammond *et al*., 2022; Hartmann *et al*., 2022) with major consequences for the ability of forests to store carbon (Johnson *et al*., 2016; Pugh *et al*., 2020). However, mortality remains the largest source of uncertainty when it comes to projecting future changes in forest structure and dynamics (Hubau *et al*., 2020; McDowell *et al*., 2020; Pugh *et al*., 2020). This is because tree mortality is an infrequent and stochastic processes that is inherently hard to capture using field inventories. Previous efforts to use remote sensing to address this challenge have mostly focussed on canopy‐scale disturbances, tracking changes in canopy cover or gap dynamics (Chambers *et al*., 2013; Senf *et al*., 2018; Cushman *et al*., 2021, 2022). A major advantage of our approach is the ability to track mortality and crown dieback at the individual tree level across entire landscapes (Figs 2c, 6c). This not only allows direct comparisons with field data but also provides an intuitive and robust way to scale up from individuals to community‐level patterns (Fig. 6).

Across the landscape, average rates of tree mortality were 1.1% yr^−1^, which is consistent with baseline rates documented in other forest types (Lines *et al*., 2010; Ruiz‐Benito *et al*., 2013; Stovall *et al*., 2019; Esquivel‐Muelbert *et al*., 2020; Hartmann *et al*., 2022). As is typically observed, mortality was highest for small trees and then declined sharply as trees grew larger (Fig. 2c). However, we did not observe a clear U‐shaped pattern of mortality, where survival rates decline in the largest size classes due to greater exposure to disturbances such as wind, lightning and drought (Coomes & Allen, 2007; Lines *et al*., 2010; Coomes *et al*., 2012; Stovall *et al*., 2019). Instead, mortality plateaued at *c*. 0.7–0.9% yr^−1^ in medium‐ and large‐sized trees, a pattern that has been reported previously in other Mediterranean woodlands (Ruiz‐Benito *et al*., 2013).

When growth and mortality rates were integrated and converted into aboveground biomass gains and losses, differences between size classes became even more apparent (Fig. 4). Similar to previous work, we found that most of the aboveground biomass is stored in a relatively small number of large trees (Fig. 4a), although this disparity is not as pronounced as in tropical rainforests where the largest 1% of trees can exceed 60–70% of the total standing biomass (Lutz *et al*., 2018). Surviving trees in the largest size classes also contributed most to aboveground biomass gains (green bars in Fig. 4b), as for a big tree even a small increase in height and/or crown size translates into large gains in terms of biomass (Stephenson *et al*., 2014; Jucker *et al*., 2017). However, these gains were partially or even completely offset by progressively greater losses in biomass associated with the mortality and dieback of large trees (brown bars in Fig. 4b), so much so that trees in top 10% for size actually lost considerably more biomass than they gained over the course of the study. Instead, it was mid‐sized trees – where rates of crown expansion peaked and mortality was at its lowest (Fig. 2) – that contributed most to net gains in live aboveground biomass across the landscape. This underscores the importance of being able to partition biomass gains and losses across size‐structured populations in order to fully understand their dynamics (Piponiot *et al*., 2022; Zuidema & van der Sleen, 2022; Yu *et al*., 2024).

### Linking tree demography to vegetation dynamics and 3D structure

Rates of tree growth and mortality varied noticeably across the landscape in relation to both competitive environment and topography (Fig. 6), the effects of which were similar in magnitude to those of tree size (Fig. 5). In terms of topography, trees were more likely to have faster rates of vertical and horizontal crown growth, as well as higher likelihood of survival, in areas of the landscape that retain more water (high TWI). This is what we would expect in a semi‐arid ecosystem such as the GWW, where water is likely the primary limiting factor to tree growth, recruitment and survival (Boisvenue & Running, 2006). It also helps explain previous work showing how vegetation structural attributes such as height, cover and biomass often vary considerably in response to seemingly subtle changes in terrain elevation and slope, typically decreasing from valley bottoms to ridges (Colgan *et al*., 2012; Swetnam *et al*., 2017; Jucker *et al*., 2018; Muscarella *et al*., 2020). The ability of ALS to concurrently map both the 3D structure of the vegetation and the underlying terrain is a major asset of this technology for ecological research (Jucker *et al*., 2018; Muscarella *et al*., 2020).

In terms of the effects of local competition, faster rates of tree growth in more densely vegetated areas were balanced out by noticeably greater risk of mortality (Fig. 5f). This effect of neighbourhood crowding on tree mortality is consistent with previous work showing that competition for light, water and nutrients are among the primary drivers of mortality in forests, especially in smaller sized trees (Coomes *et al*., 2012; Ruiz‐Benito *et al*., 2013). The fact that height growth also increased in taller neighbourhoods matches our understanding of how trees shift their investment towards vertical growth in dense stands (Jucker *et al*., 2024; Laurans *et al*., 2024). However, somewhat counter‐intuitively we found that crown expansion rates were also greater in dense stands. The fact that trees in denser stands grew faster both vertically and laterally suggests that something else in addition to (or instead of) competition might be speeding up canopy dynamics in these taller patches of woodland. One possible explanation would be the patchy distribution of specific soil and bedrock types that in turn provide the conditions for trees to form locally dense stands characterised by faster rates of growth and mortality – again pointing to topography as an overarching driver of landscape‐scale variation in woodland structure and dynamics.

More generally, capturing how tree demographic rates are shaped by both topography and local competitive environment allowed us to shed light on the processes that give rise to the clustered distribution of vegetation across the landscape (Fig. 1). These spatial patterns are a hallmark of semi‐arid ecosystems (Rodriguez‐Iturbe *et al*., 2019; Veldhuis *et al*., 2022) and are increasingly used as indicators of their resilience to disturbance and climate change (Kéfi *et al*., 2007, 2024; Veldhuis *et al*., 2022). For instance, in the GWW, the patchy distribution of trees in old‐growth stands can help prevent the spread of stand‐replacing wildfires (Gosper *et al*., 2018; Jucker *et al*., 2023). But how these vegetation spatial patterns emerge as a result of variability in tree growth and survival rates across heterogeneous landscapes is not well‐established. In this regard, our work provides a starting point for linking demography to vegetation spatial patterns through the integration of empirical data, remote sensing and models (Jucker, 2022).

Tracking the fate of individual trees through time also allowed us to partition aboveground biomass dynamics into gains and losses, and explicitly link these back to changes in vegetation 3D structure. Our results indicate that over the 9‐yr period of this study, the net balance between biomass gains from tree growth and losses due to mortality was positive across the landscape (Fig. 4). This strongly suggests that in the absence of large wildfires, these old‐growth obligate‐seeder woodlands can continue to operate as net carbon sinks even when several centuries old (Gosper *et al*., 2018). These positive biomass trends closely mirrored those in canopy cover and volume captured directly from the CHMs, both of which also increased across the landscape (Fig. 6e,f). Similar patterns of increasing woody cover have been reported in African savannas and woodlands over recent decades (Venter *et al*., 2018; Zhao *et al*., 2024), where increasing CO_2_ concentrations are believed to be shifting the balance in favour of trees overs C_4_ grasses.

Somewhat surprisingly, we found that net biomass change across the study site was positive even though a large disturbance event occurred sometime between 2012 and 2021 (Fig. 6). This event affected a strip *c*. 0.6 km^2^ in size (2.4% of the landscape) that roughly follows the course of a creek bed (Fig. 1b), within which a marked decrease in canopy cover, height and biomass were observed. Specifically, we estimate that *c*. 2336 Mg of biomass were lost due high rates of tree mortality in the affected area, *c*. 3.5% of the total woody biomass found across the entire site in 2012. As no large fires or human‐related disturbances occurred in this period, this disturbance event was most likely the result of a tornado, which are common in the aftermath of tropical cyclones tracking inland from the north‐western Australian coast. This provides clear evidence that other disturbances aside from stand‐replacing fires can play an important role in shaping the structure and dynamics of these semi‐arid woodlands (Yates *et al*., 1994).

### Tracking forest dynamics one tree at a time from above – future opportunities and challenges

From a methodological standpoint, an important contribution of our study is the development of an improved and more flexible implementation of the widely used Dalponte & Coomes (2016) tree crown segmentation algorithm. By incorporating a two‐stage crown delineation routine, we were able to substantially improve the retrieval of large trees that make up most of the aboveground biomass (Fig. 4a; Lutz *et al*., 2018). Stand‐level estimates of tree density, basal area and aboveground biomass derived from our ALS‐segmented trees closely matched independent observations from field data, suggesting our results are robust. Nonetheless, there are several ways in which our approach could be built on going forward. An obvious one would be to use complementary remote sensing approaches, such as canopy spectroscopy, to map the traits, functional groups or even species of the individually segmented crowns (Asner *et al*., 2015; Marconi *et al*., 2022; Beloiu *et al*., 2023). This would reveal how growth and mortality rates vary among dominant species, thus building a more complete picture of the dynamics of these ecosystems. From a demography perspective, the missing piece of the puzzle is quantifying tree recruitment. This would require more frequent ALS acquisitions to avoid underestimating recruitment rates due to high mortality of small trees. But even then, estimating recruitment and understory dynamics from remote sensing will always remain a major challenge, especially in denser forests where seedlings recruit in the understory. For this, leveraging data from ground monitoring networks will continue to be essential.

More generally, there are several important sources of uncertainty and limitations associated with mapping individual trees from ALS that deserve careful consideration. First and foremost, even in an open canopy system such as the GWW, individual tree detection using ALS is not perfect (Cao *et al*., 2023), nor is the matching of segmented crowns across time periods. Consequently, while repeat ALS scans provide an opportunity to track the dynamics of large numbers of trees across whole landscapes, what we obtain is a sample of the population with its own set of biases (e.g. with respects to tree size, but also dependent on a tree's immediate neighbourhood; Cao *et al*., 2023). Even when we do correctly identify trees in ALS scans, there is still the issue accurately measuring their vertical and horizontal growth rates. Differences in sampling density and flight configuration across ALS scans can severely affect the retrieval of canopy attributes (Zhang *et al*., 2024), although we can effectively mitigate these sources of uncertainty by using robust CHM algorithms and statistically correcting for differences in sampling density across ALS scans (Fischer *et al*., 2024; Jackson *et al*., 2024), as we have done here. Finally, if we want to convert changes in tree height and crown size into units of biomass, we need to rely on allometric equations that relate a tree's crown dimensions to its mass (Jucker *et al*., 2017). Not only do these equations carry larger uncertainty than traditional biomass models based on stem diameters, but the field data used to calibrate them may also systematically differ from ALS‐based estimates of tree height and crown size (Terryn *et al*., 2024). All these sources of error are minimised when working in open canopy systems with flat terrain dominated by relatively large, single‐stemmed trees (Brandt *et al*., 2020) – which is a key reason why we chose the GWW for our study. But at the same time, this also means our results represent a best‐case scenario for how well these individual‐based methods currently work.

It is only through the integration of novel remote sensing approaches with long‐term field records that we will be able to develop a better understanding of how forests are responding to rapid global change. There is great potential for harnessing these complementary data streams to build better individual‐based models of forest dynamics (Lines *et al*., 2022). Traditionally, these models have been parameterised and validated against field data, relying heavily on stem diameter measurements that only indirectly reflect whole‐plant growth and competitive environment. Using repeat ALS data to map large numbers of individual trees can allow us to capture population‐level trends in growth and mortality in a much more comprehensive way, which is crucial for calibrating and validating forest models. In this regard, our study provides a blueprint for mobilising increasingly available high‐resolution remote sensing data to track the spatial and temporal dynamics of open canopy forests so that we may better understand how they will respond in an increasingly warmer and more fire‐prone future (Jucker *et al*., 2023).

## Competing interests

None declared.

## Author contributions

TDJ and SMP conceived the idea for the project. KZ coordinated the 2021 ALS survey. RB led the processing and analysis of the data, with assistance from TJ, TDJ and FJF. RB and TJ wrote the first draft of the manuscript, with all co‐authors contributing substantially to revisions.

## Supporting information


**Fig. S1** Map of the study region.
**Fig. S2** Crown detection accuracy of alternative tree segmentation algorithms.
**Fig. S3** Crown diameter to tree height allometries.
**Fig. S4** Accuracy of alternative implementations of the *dalponte2016* algorithm.
**Fig. S5** Comparison of mean and maximum height change estimates.
**Fig. S6** Variation in airborne laser scanning pulse density across the study area.
**Fig. S7** Spatial autocorrelation in generalised additive model residuals.
**Methods S1** Masking out mulga scrub habitat.
**Methods S2** New implementation of the *dalponte2016* segmentation algorithm.
**Table S1** Comparison of alternative tree segmentation algorithms.
**Table S2** Generalised additive model output summaries.
Please note: Wiley is not responsible for the content or functionality of any Supporting Information supplied by the authors. Any queries (other than missing material) should be directed to the *New Phytologist* Central Office.

## Data Availability

The 2012 ALS data used in this study are openly available through the TERN data portal (https://portal.tern.org.au). All other data and code underpinning the results of this paper are publicly archived on Zenodo and can be accessed here: doi: 10.5281/zenodo.13871110. This includes: (1) 2012 and 2021 canopy height models (0.5‐m resolution, GeoTIFF format), (2) the digital terrain model and topographic wetness index raster (5‐m resolution, GeoTIFF format), (3) a data frame containing the tree‐level demographic data, along with covariates included in the statistical models (CSV format) and (4) R code to replicate the analyses presented in the main text.
